# Synchronous Primary Lung Cancer: An Unusual Triple Presentation

**DOI:** 10.7759/cureus.86620

**Published:** 2025-06-23

**Authors:** Diogo Fernandes, Joana Ip, Juliana Filipe, Rita Barata, Patricia Winckler

**Affiliations:** 1 Radiology, Instituto Português de Oncologia de Coimbra Francisco Gentil, Coimbra, PRT; 2 Radiology, Champalimaud Clinical Centre, Lisbon, PRT; 3 Pathology, Champalimaud Clinical Centre, Lisbon, PRT; 4 Thoracic Surgery, Champalimaud Clinical Centre, Lisbon, PRT; 5 Oncology, Champalimaud Clinical Centre, Lisbon, PRT

**Keywords:** chest, ct, lung cancer, sbrt, surgical treatment, synchronous primary tumors

## Abstract

This case report describes the case of a 75-year-old woman, a former smoker, who began to experience progressive fatigue and shortness of breath. She underwent some tests that did not reveal any significant changes. Still, as the complaints persisted, a computed tomography (CT) scan of the chest was performed, which showed the existence of three suspicious lung lesions, two in the right lung (upper lobe and middle lobe) and one in the left lower lobe. The three lesions were biopsied, which revealed lesions that were positive for CK7 and TTF-1 and negative for p40, suggestive of lung adenocarcinoma. The case was discussed in a multidisciplinary meeting, and the existence of synchronous multiple primary lung cancers (SMPLC) was assumed; hence, it was decided to perform a right bilobectomy and left lower lobe stereotactic body radiation therapy (SBRT). Histology revealed that all the tumors were different, and this was a case of three SMPLC. Despite their rarity, synchronous lung tumors are increasingly diagnosed, bringing with them numerous diagnostic and therapeutic dilemmas, since their overall survival is superior to that of multiple lung metastases from lung carcinoma.

## Introduction

Lung cancer is the most diagnosed cancer and the primary cause of cancer mortality worldwide, accounting for 18% of all cancer deaths [1]. Lung cancer is divided into two main histologic types: non-small cell lung cancer (NSCLC), responsible for 84% of cases, and small cell lung cancer (SCLC), responsible for 13% of cases [2]. Continued smoking remains the principal cause of lung cancer, contributing to approximately 80% of the cases and 80-90% of lung cancer deaths [3].

When diagnosing lung cancer, the evidence of multiple pulmonary lesions is increasing because of the great development of detection techniques such as computed tomography (CT) and positron emission tomography (PET) [4]. This leads to management dilemmas and the need to distinguish between the presence of metastatic disease from one primary lung cancer and multiple primary lung cancers (MPLC) with distinct clonal origin [5]. MPLC is classified as synchronous (SMPLC) when occurring at the same time or metachronous when occurring at different times [6]. The diagnosis interval between two malignant lung cancers is generally ≤6 months for synchronous cancers, although a different interval should be considered for metachronous cancers [7].

The distinction between SMPLC from both lung metastasis of primary lung tumors and multiple lung metastases of non-lung tumors is difficult. Although distinguishing SMPLC from both lung metastases of primary lung tumors and multiple lung metastases of non-lung tumors is challenging, it is crucial for determining appropriate therapeutic management and prognosis [4,8]. The average incidence of synchronous lung cancers ranges from 0.2% to 8% (3.5-14% in autopsy studies). Biopsy is crucial for characterizing each lesion, enabling genomic analysis, and allowing for the most effective treatment for each cancer, with significant prognostic implications [8].

Despite the advances in the detection and treatment of lung cancer, the prognosis remains poor, with a global five-year survival rate of less than 20%. Overall, the prognosis of SMPLC is better than that of single primary lung cancer, reaching 77% in some studies, but worse than metachronous lung cancers [9].

## Case presentation

This paper presents the case of a 75-year-old woman, an ex-smoker with 16 pack-years, with Hashimoto thyroiditis, without any other relevant personal history, and only medicated with levothyroxine for the thyroiditis. From the end of 2023, the patient reported increasing fatigue, which evolved to affect minor daily activities by the second quarter of 2024. She decided to visit her primary care physician after that, being submitted to some blood workups, cardiac exams, and chest X-rays, which did not reveal any significant findings.

**Figure 1:**
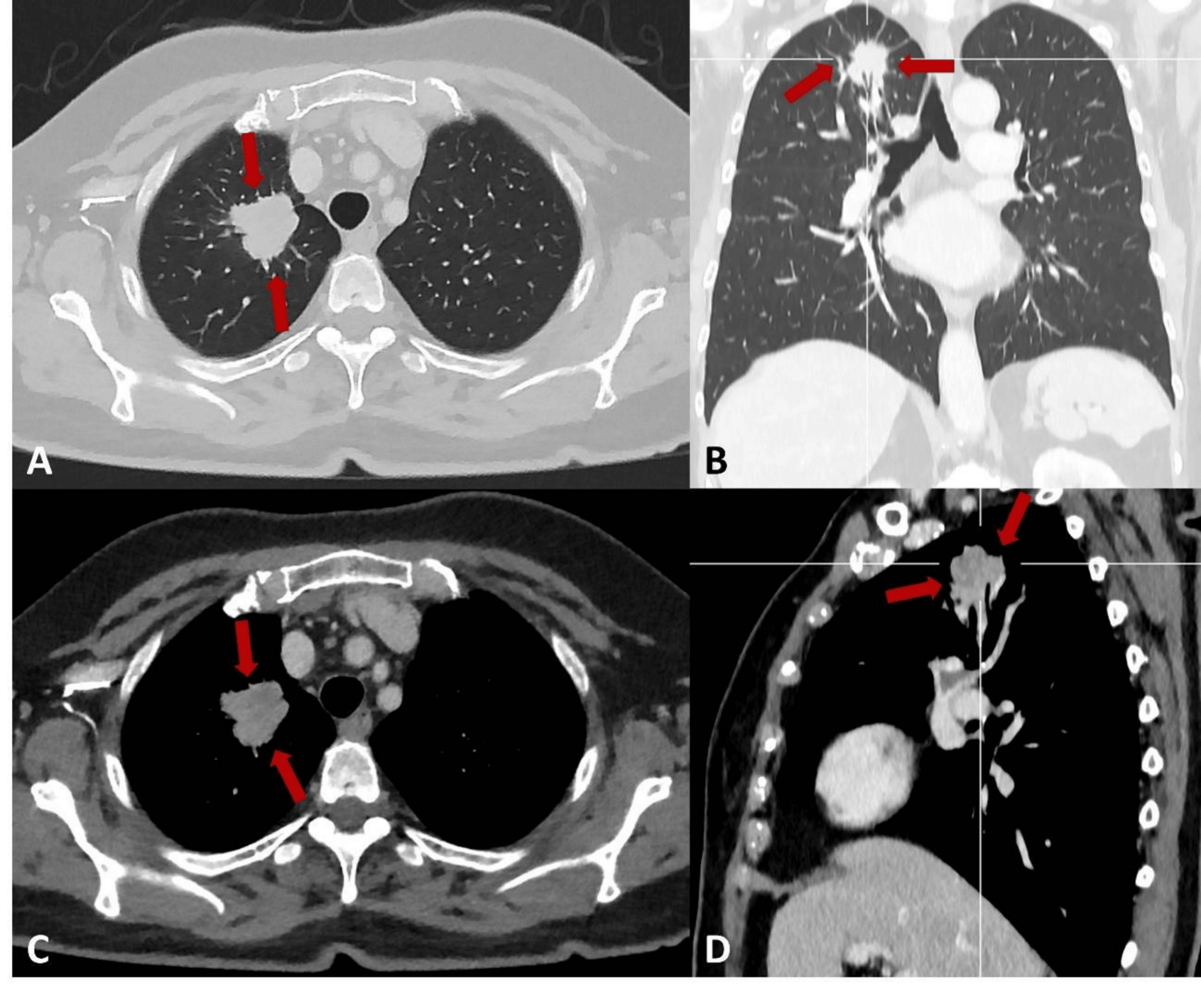
Chest CT showing a solid nodule with spiculated contours (red arrow) in the apical segment of the right upper lobe. (A) Lung window axial plane. (B) Lung window coronal plane. (C) Soft tissue window with contrast axial plane. (D) Soft tissue window with contrast sagittal plane. CT: computed tomography

**Figure 2:**
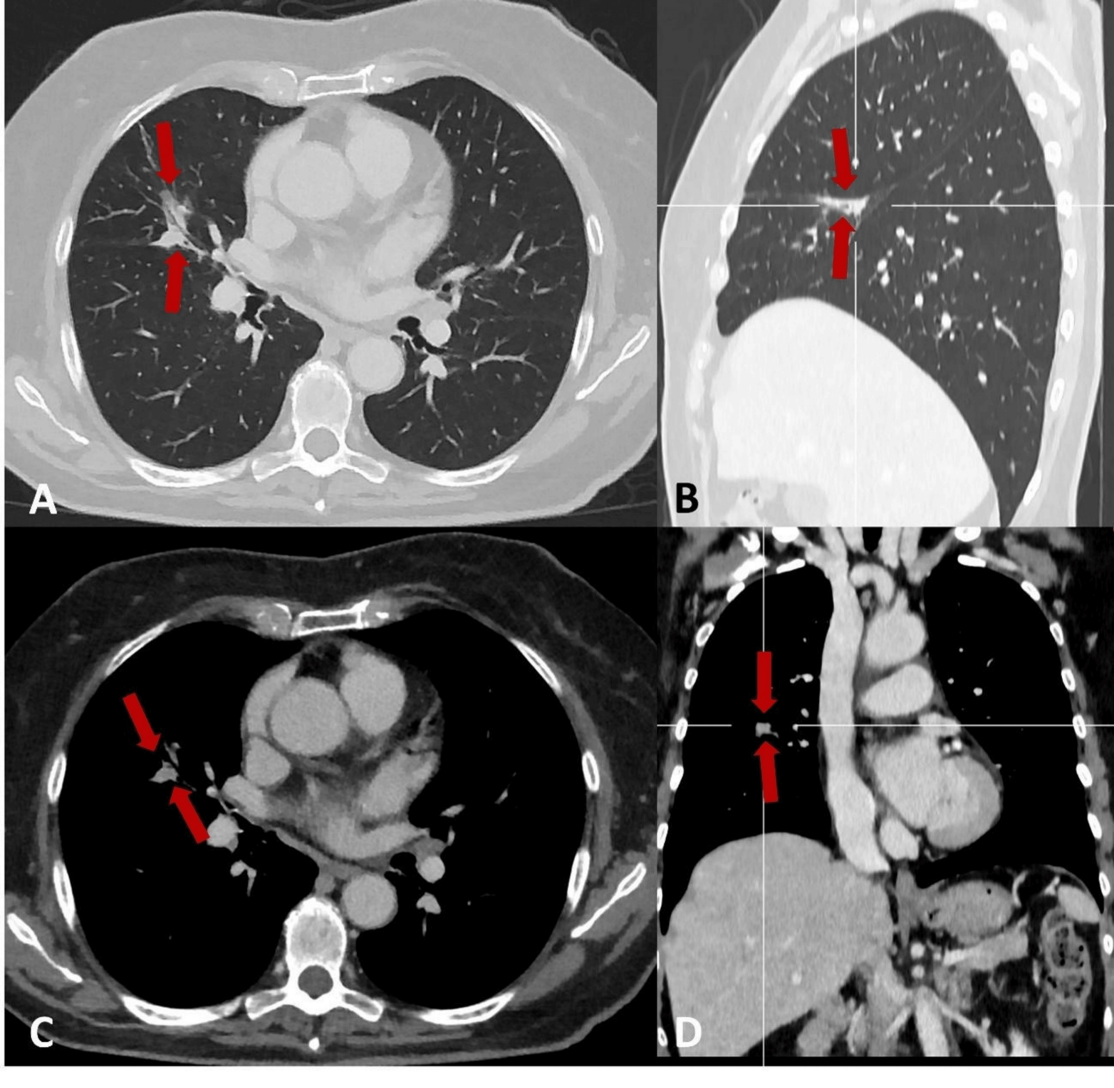
Chest CT showing an ill-defined solid area (red arrow) in the middle lobe. (A) Lung window axial plane. (B) Lung window sagittal plane. (C) Soft tissue window with contrast axial plane. (D) Soft tissue window with contrast coronal plane. CT: computed tomography

**Figure 3:**
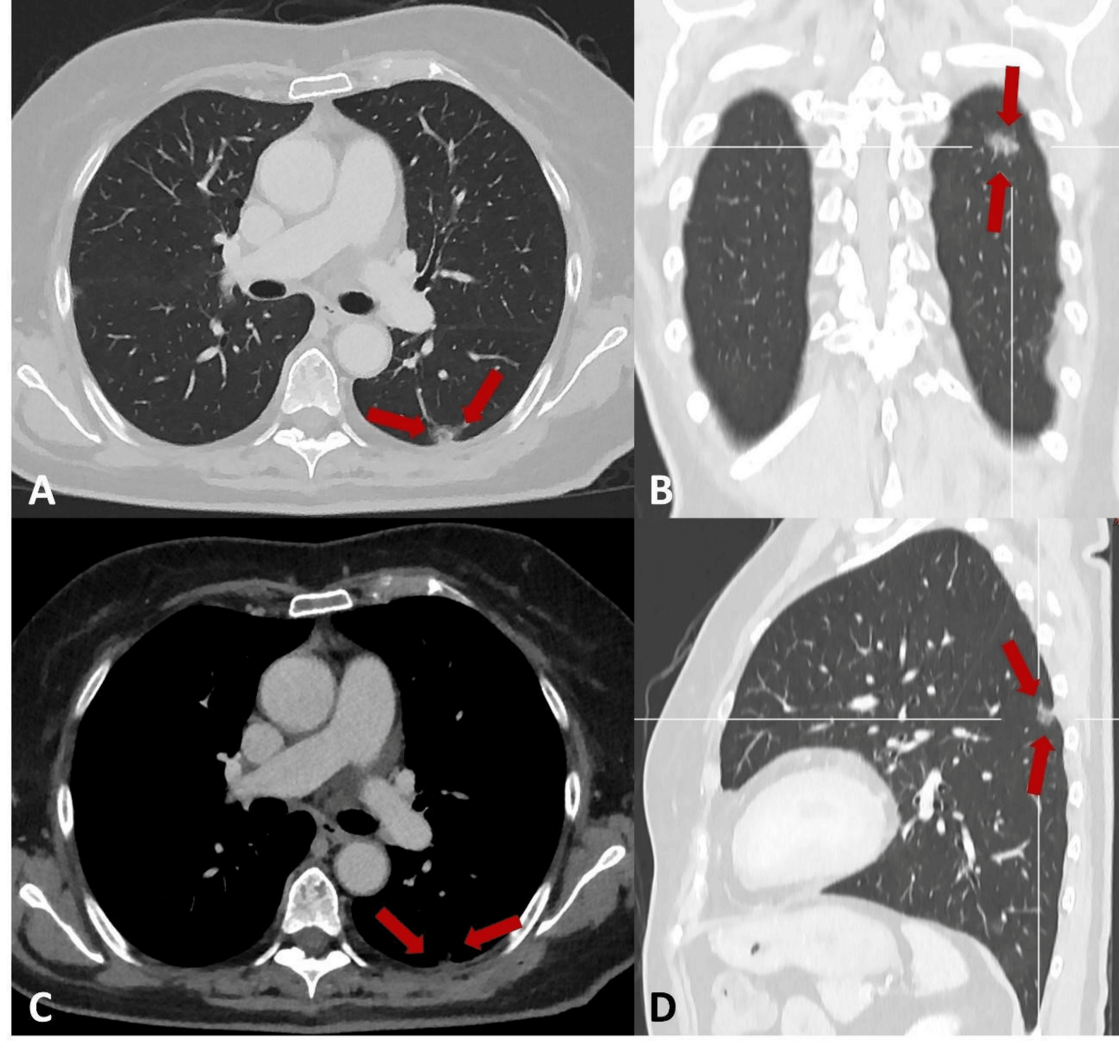
Chest CT showing a subsolid area in the upper segment of the left lower lobe (red arrow), predominantly ground-glass and ill-defined. (A) Lung window axial plane. (B) Lung window coronal plane. (C) Soft tissue window with contrast axial plane. (D) Lung window sagittal plane. CT: computed tomography

As the changes persisted, the primary care physician requested a CT scan, which was carried out in December 2024. It revealed suspicious findings in the lungs: a solid nodule with spiculated contours in the apical segment of the right upper lobe (Figure 1), an ill-defined solid area in the middle lobe (Figure 2), and a subsolid area in the upper segment of the left lower lobe, predominantly ground-glass and ill-defined (Figure 3).

**Figure 4:**
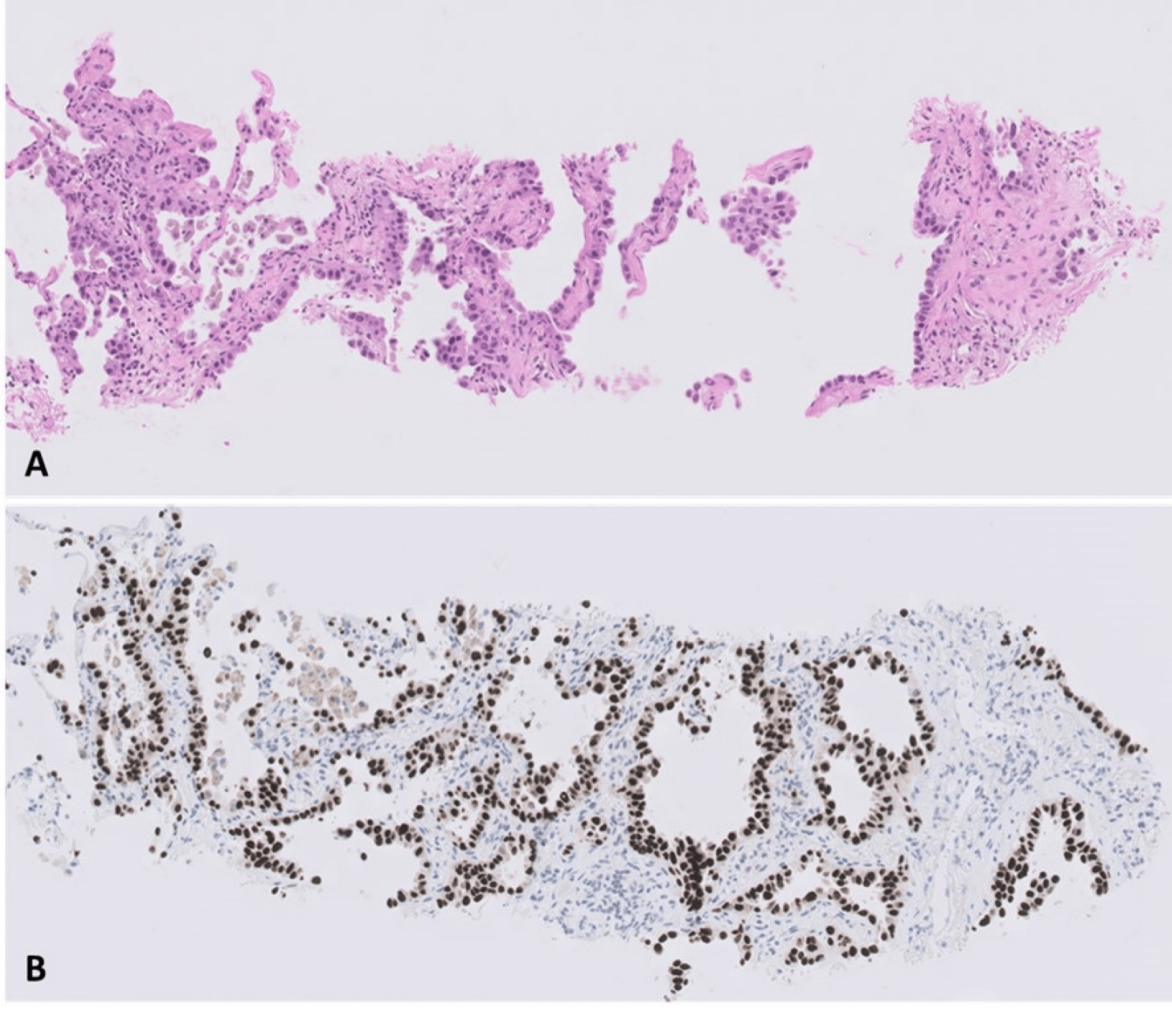
Biopsy histology from the left lower lobe lesion showing an adenocarcinoma with lepidic and acinar patterns, positive for TTF-1. (A) H&E staining. (B) Immunohistochemical staining for TTF-1.

The patient was then admitted to the Champalimaud Clinical Centre (CCC) and discussed in a multidisciplinary meeting. Because the three lesions present different characteristics on CT, raising the possibility that they are synchronous tumors, it was decided to perform a biopsy on the three suspicious lesions. This revealed the existence of three lung cancers, of the adenocarcinoma type, in the right upper lobe and middle lobe with an acinar pattern and in the left lower lobe with a lepidic and acinar pattern (Figure 4).

Mediastinal staging was performed with PET-CT and endobronchial ultrasound (EBUS). The results were negative for mediastinal involvement, which reinforced the possibility that these were three synchronous tumors. The patient was discussed again in a multidisciplinary meeting, and it was decided to perform a right bilobectomy and stereotactic body radiation therapy (SBRT) on the lesion in the left lower lobe.

**Figure 5:**
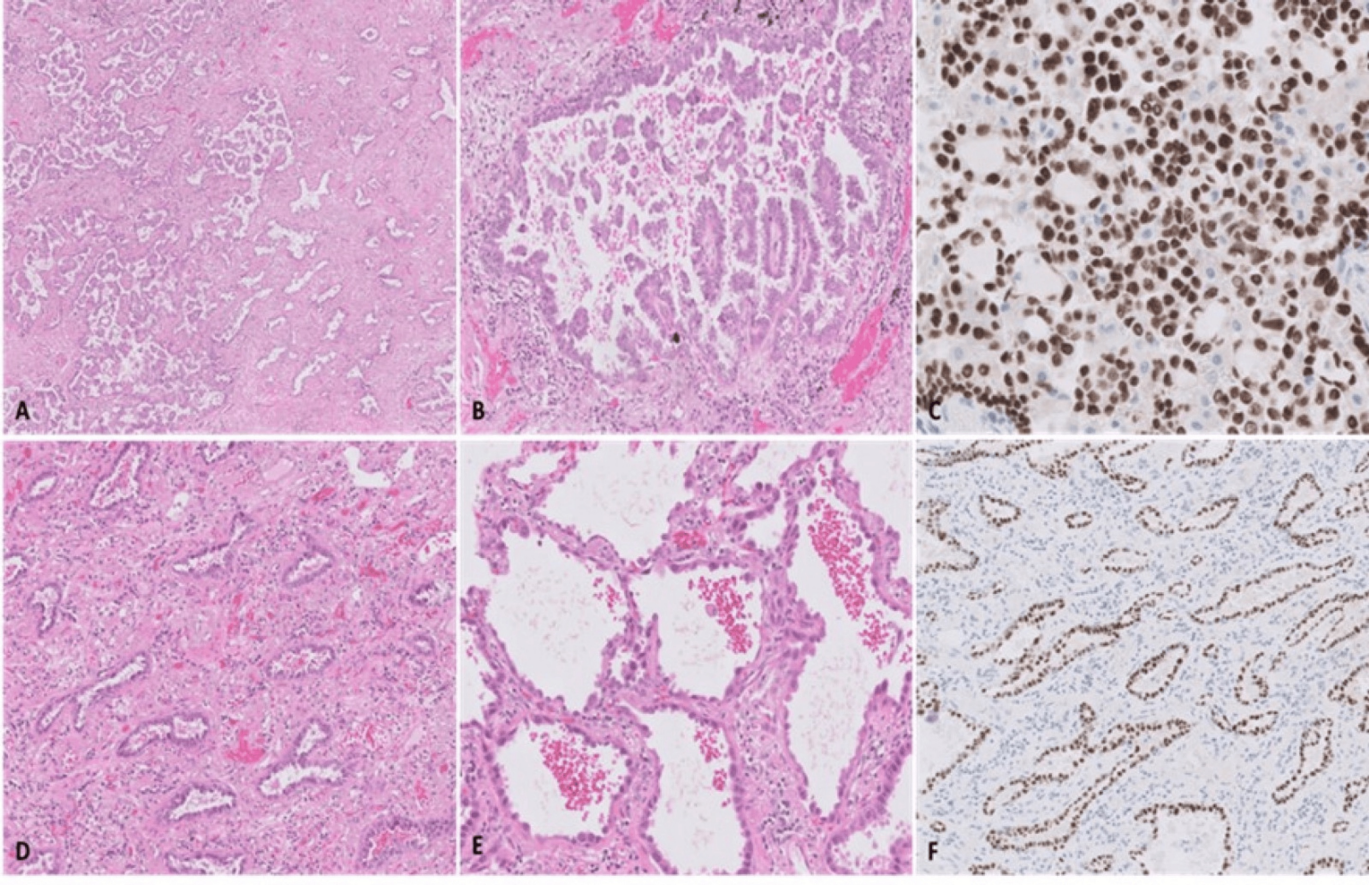
Bilobectomy histology showing that the right upper lobe adenocarcinoma (A, B, and C) is morphologically different from the middle lobe adenocarcinoma (D, E, and F). On the right upper lobe, the adenocarcinoma showed predominantly a micropapillary pattern, with also acinar and papillary patterns. On the middle lobe, the adenocarcinoma showed predominantly an acinar pattern with areas with a lepidic pattern. Both tumors were TTF-1 positive. (A, B, D, and E) H&E staining. (C and F) Immunohistochemical staining for TTF-1.

Surgery was performed, and after histological analysis of the right upper lobe and middle lobe, it was concluded that we were dealing with three completely distinct adenocarcinomas: in the right upper lobe, a non-mucinous adenocarcinoma with a predominantly micropapillary pattern (40% micropapillary, 25% papillary, 25% acinar, and 10% lepidic) that is poorly differentiated (G3), positive for TTF-1, and negative for PAX8, pT2aN1 (Figure 5); in the middle lobe, a non-mucinous adenocarcinoma with predominantly acinar pattern (70% acinar and 30% lepidic) that is moderately differentiated (G2) and TTF-1 positive, pT1bN0 (Figure 5); and in the left lower lobe, a lepidic and acinar adenocarcinoma that is CK7+ and TTF1+ (Figure 4).

In the post-surgical evaluation, the patient reports progressive improvement, with reduced asthenia, and is undergoing respiratory rehabilitation.

## Discussion

Lung cancer is the most common and deadly cancer worldwide, most common among men, and tobacco smoking accounts for over 80% of lung cancer cases and is the leading preventable cause of death worldwide [3].

With the advances in the diagnosis of lung cancer, 16-28% of the patients with lung cancer had other lung nodules, creating confusion in the diagnostic process to determine whether these nodules are primary tumor metastasis, MPLC, or benign lesions. For that reason, the incidence of SMPLC is increasing, reaching 20% in some series [10].

The diagnosis of SMPLC requires tissue sampling of all tumors because nodules in the ipsilateral lung are often regarded as metastasis and also because multiple adenocarcinomas are reported to account for as much as 50% or greater of SMPLC [11]. However, even for histological experts, making an accurate diagnosis can be difficult, and genomic testing with next-generation sequencing (NGS) is often necessary. In one study, histology alone allowed for the differentiation between intrapulmonary metastasis and SMPLC in 65% of patients, while the use of NGS provided a conclusive diagnosis in 94% of cases [12].

The staging of a patient presenting with MPLC is complex and must be conducted meticulously when a potentially curative resection is being considered. In addition to CT, the patient should undergo brain magnetic resonance imaging (MRI) to rule out brain metastases, as well as fluorodeoxyglucose positron emission tomography (FDG-PET) to assess for extrathoracic and lymph node metastases. The absence of mediastinal lymph node involvement and distant metastases is essential for the diagnosis of SMPLC [10].

Distinguishing between SMPLC and pulmonary metastases is crucial for determining the most appropriate treatment strategy. In general, long-term survival is higher in patients with SMPLC who undergo surgical resection; therefore, surgery is considered a key treatment option in eligible patients [13].

In the case presented, the patient had three primary lung tumors: one in the right upper lobe, one in the middle lobe, and one in the left lower lobe. Although the patient had sufficient pulmonary reserve to undergo surgical resection of all three tumors, the aggressiveness of the lesions necessitated an initial right bilobectomy. A second surgery was planned after the patient's recovery.

However, due to the anticipated delay in scheduling a second surgical procedure compared to the shorter waiting time for SBRT, the treatment plan was revised. It was decided to proceed with a right bilobectomy followed by SBRT. This approach was considered to offer the greatest benefit to the patient.

Video-assisted thoracoscopic surgery (VATS) is a feasible and safe approach that offers significant benefits for patients with SMPLC. However, most existing studies and case reports focus on cases involving only two synchronous lung tumors [14]. In the present case, three synchronous tumors were identified, but surgical resection of all lesions was not feasible.

A Chinese surgical group has proposed that, in the absence of contraindications, surgical resection should remain the primary treatment modality for SMPLC. Their approach emphasizes both the preservation of functional lung parenchyma and the complete, effective resection of malignant lesions. When complete resection of all tumors in a single procedure is not feasible, priority should be given to removing the cancer with the poorest prognostic features [14].

In our patient's case, a right bilobectomy was performed to remove the tumors with the worst prognosis, followed by SBRT to treat the left lower lobe tumor. This approach allowed for the timeliest intervention while offering the most favorable prognosis for the patient.

It is now well established that the overall survival of patients with MPLC is superior to that of patients with advanced-stage tumors, including those with locally recurrent or metastatic disease. Notably, overall survival does not appear to be significantly influenced by whether the histological subtypes of MPLC are identical or distinct, nor does it differ between unilateral and bilateral presentations [6].

MPLC is further subclassified into synchronous and metachronous forms. Patients with SMPLC tend to exhibit slightly reduced overall survival compared to those with metachronous MPLC, primarily due to the absence of a tumor-free interval between diagnoses in the synchronous group [6]. The therapeutic strategy adopted in this case was designed to optimize the patient's long-term survival prospects, balancing timely intervention with oncologic efficacy.

Patients diagnosed with lung cancer are at an elevated risk of developing additional primary lung malignancies, necessitating lifelong surveillance. Early detection of a second primary carcinoma may enable radical resection in appropriately selected patients or facilitate timely initiation of palliative therapies such as radiotherapy and/or chemotherapy [4].

While various guidelines have been proposed, there is ongoing debate regarding the extent to which intensive surveillance directly contributes to improved survival outcomes. Nonetheless, the most widely adopted approach involves more frequent reassessment during the initial years following treatment, typically every 3-6 months, through clinical evaluation and chest CT, with progressively increasing intervals thereafter [15]. Smoking cessation remains a critical component of risk reduction for subsequent primary tumors, and referral for specialized smoking cessation support is strongly recommended [15].

Our patient is currently undergoing close post-treatment surveillance and has already attended several follow-up evaluations. The first surveillance CT scan is scheduled for the second quarter of 2025.

## Conclusions

In patients with the diagnosis of lung cancer and with more than one lesion, the possibility of SMPLC should be considered, and histopathological sampling should be performed for each lesion separately because the prognosis of MPLC is better than that of lung cancer patients with intrapulmonary metastases. It is essential to accurately exclude the possibility of metastases and provide the best treatment care, as aggressive surgery strategies are the best option for suitable patients.
